# Validation of high temporal resolution spiral phase velocity mapping of coronary artery blood flow against Doppler flow wire

**DOI:** 10.1186/1532-429X-17-S1-O78

**Published:** 2015-02-03

**Authors:** Jennifer Keegan, Claire E Raphael, Robin Simpson, Kim H Parker, Ranil de Silva, Carlo Di Mario, Sanjay K Prasad, David Firmin

**Affiliations:** 1 Royal Brompton Hospital, London, UK; 2 Imperial College, London, UK; 3 University of Freiburg, Freiburg, Germany

## Background

The temporal pattern of coronary artery flow velocity through the cardiac cycle provides important information about coronary haemodynamics. The ‘gold standard' for coronary artery velocity assessment is the Doppler flow wire which is inserted directly into the coronary artery under X-ray fluoroscopic guidance, which is invasive, uses ionising radiation and has a small but significant risk of serious complication. In this study, we perform an in vivo comparison of coronary blood flow velocities measured with breath-hold high temporal resolution spiral phase velocity mapping and Doppler flow wire measurements.

## Methods

Breath-hold proximal coronary artery velocity maps were acquired in 5 left anterior descending (LAD) and 3 right coronary arteries (RCAs) in patients who had undergone invasive Doppler flow wire assessment as part of a clinical assessment. Acquisition parameters were: pixel size 1.4 x1.4 mm (reconstructed to 0.7 x 0.7 mm) x 8 mm, true temporal resolution 19 ms, 1-1 water excitation (WE), 17 cardiac cycle breath-hold, velocity sensitivity 30-50cm/s, retrospective ECG gating (reconstructed 50 phases). Extraction of MR coronary artery blood flow velocity-time curves was performed using a custom MATLAB program which tracked the motion of the vessel over the cardiac cycle and corrected for the through-plane motion of the vessel using a region of adjacent tracked myocardium (LAD) or a region of surrounding epicardial fat (RCA) from a separately acquired fat-excitation acquisition. For each vessel, the mean through-plane motion-corrected MR velocities at all timepoints were plotted against the equivalent flow wire velocities. If necessary, the profiles were first time-shifted to take into account small differences in data triggering between the two techniques. The correlation between the two profiles was assessed using simple linear regression analysis.

## Results

**Figure 1:**
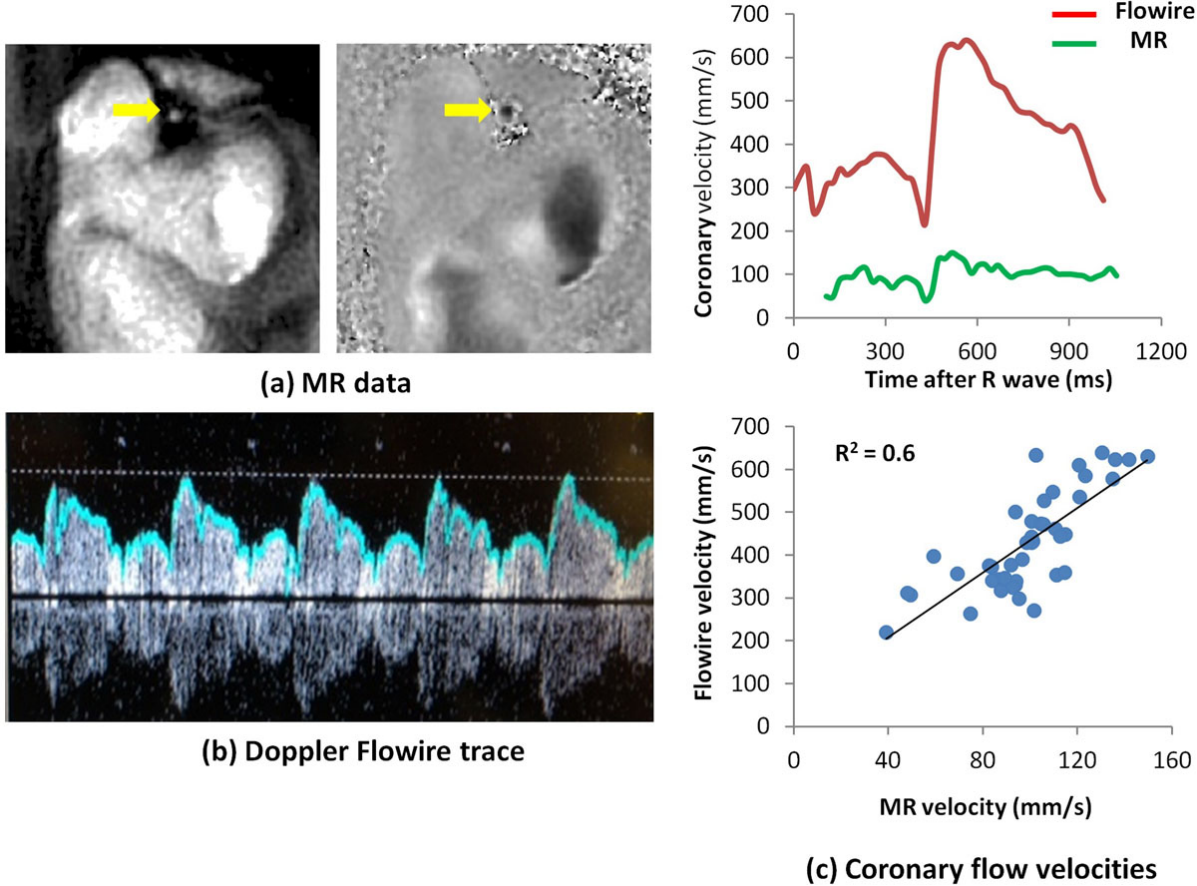
Example early diastolic LAD (arrows) magnitude image and velocity map from a high temporal resolution spiral phase velocity mapping acquisition (a), together with the corresponding Doppler Flowire trace (b). A plot of MR and Flowire coronary blood flow velocity against time in the cardiac cycle is shown in (c), together with a plot of Flowire velocities against MR velocities.

**Figure 2:**
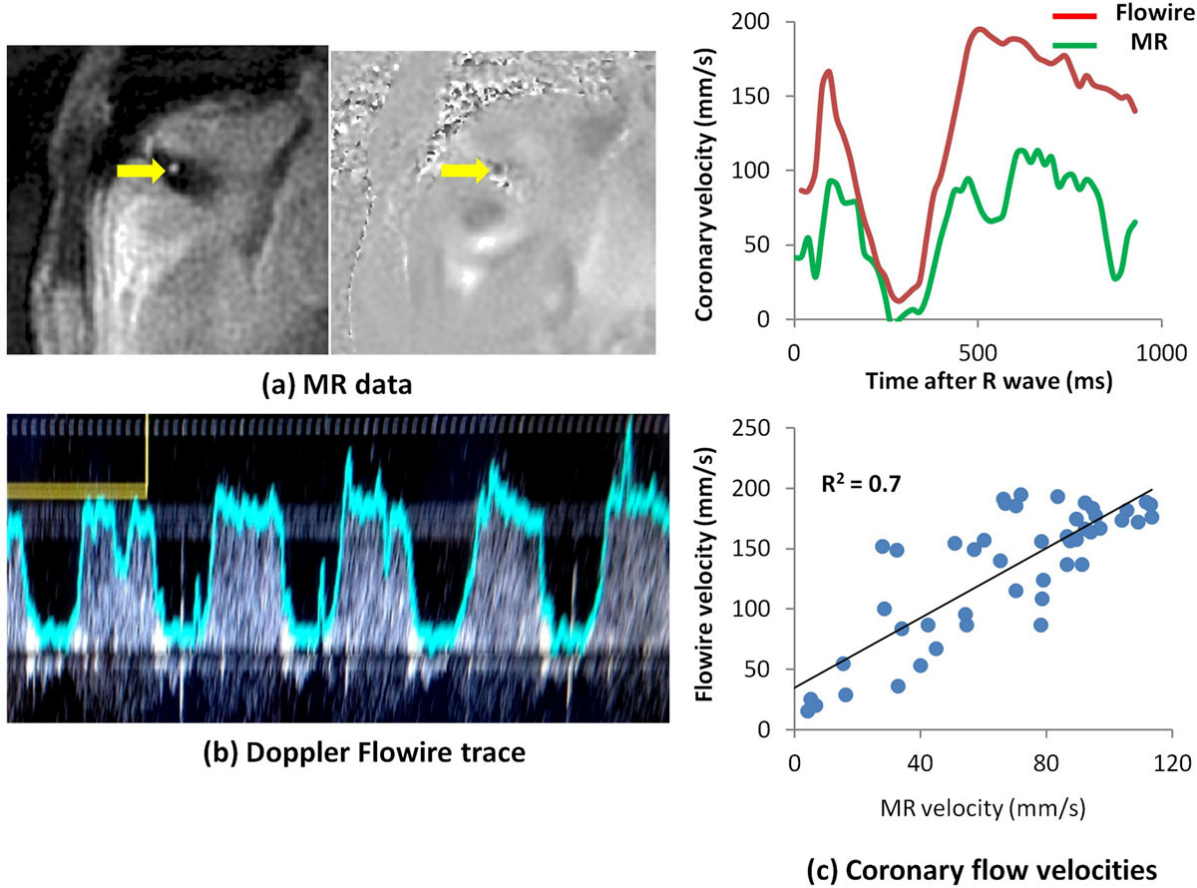
Example early diastolic RCA (arrow) magnitude image and velocity map from a high temporal resolution spiral phase velocity mapping acquisition (a), together with the corresponding Doppler Flowire trace (b). A plot of MR and Flowire measured coronary blood flow velocities against time in the cardiac cycle is shown in (c), together with a plot of the Flowire velocities against the MR velocities.

Figures 1 and 2 show example LAD and RCA studies. On average, the MR measured velocities were 66% less than the Flowire velocities (SD 9%, range: 50% - 78%). The correlation between the MR and flow wire data, however, was high, with R^2^ values ranging from 0.6 - 0.95 in the 8 vessels studied (mean R^2^ = .80).

## Conclusions

As expected, absolute measures of velocity obtained with the Doppler flow wire are higher than MR measurements as Doppler determines the peak velocity in a small sample volume, rather than the mean velocity over the cross-sectional area of the vessel. However, temporal flow profiles measured in the proximal coronary arteries using breath-hold interleaved spiral phase velocity mapping are highly similar to those measured with Doppler flow wire. We conclude that spiral phase velocity mapping of coronary artery blood flow has the potential to assess temporal flow patterns in the proximal coronary arteries.

## Funding

British Heart Foundation clinical research training fellowship;

Wellcome Institutional Startegic Support Fund: Networks of Excellence Scheme.

